# On Partial Responsibility in Lunacy and Nervous Disorders
*This paper was read by the author before the Medico-Psychological Society of Paris, at the opening of a discussion *On Partial Responsibility.*


**Published:** 1863-10

**Authors:** Legrand du Saulle


### Art. VII.—ON PARTIAL RESPONSIBILITY IN LUNACY AND NERVOUS DISORDERS.*

By Dr. Legrand du Saulle.

In entering upon the discussion of the subject of *partial responsibility,* I do not conceal from myself either the extent or the dangers of my task. I know that I am about to handle some points in science still controverted. I know, too, that I shall come into collision here and there with sincere convictions; but in answer to the appeal which has been made by M. Alfred Maury, I fully understand that it becomes the duty of every one to render assistance in the elucidation of this matter, and I now proceed to do so. If, moreover, I have sought the dangerous honour of entering first into the lists, it is because I judged that it was not permissible to any one to hold back when a question was agitated which involved self-devotion to science.

The more I study jurisprudence and its relations to medicine, the more I perceive the great necessity of being able to determine with more precision the diagnostic signs of reason, passion, and madness. In a criminal process, in which a question in morbid psychology is debated, the judicial decision ought to be the natural consequent to the diagnosis made. All the difficulty lies there.

As interpreters of a language not understood by legal authorities, medical men express to their hearers impressions of the very highest order. We have a right to be entrusted with the duty with which we have been entrusted, and our desire should be always to fulfil that duty; but nothing is more difficult than to preserve intact a conquered territory, and already we have laid ourselves open to judicial doubt. I would not for a moment call in question the eminent services which have been rendered by science to the tribunals, during the last thirty years, for instance; but I may be allowed to say that the solution of a question has but too often borne the impress of our hesitation and embarrassment, and that the observation of our disagree-

---

* This paper was read by the author before the Medico-Psychological Society of Paris, at the opening of a discussion *On Partial Responsibil ity.*

ment has but too frequently inspired the judges with slight confidence in our opinions. We have been wishful to protect against the extreme penalties of the law, the delinquencies prompted by an unsettled brain, and our evidence has been referred to sentimental eccentricities. We have also been wishful in cases of complete destruction of volition, to preserve the life of an incurable patient, and our influence has not unfrequently been followed by an unintelligible verdict.

Blame has been cast upon medical men for having too frequently exaggerated the influence of delirium upon the will. On the other hand, blame has been cast upon legal authorities for having, in many instances, refused to make sufficient allowance for morbid suggestions. Probably there has been an excess of zeal on both sides; but the antagonism of yesterday ought to-day to be converted into a mutual understanding. The time has arrived for a reciprocal concession of opinions.

The discussion which is now opened should be firmly established upon, and in a measure constitute a branch of, scientific jurisprudence. Let us, then, construct a code of doctrine; let us extend with a less enthusiastic liberality the circle of excuses, and whenever one of us shall find ourselves summoned before a court of justice to give evidence with respect to slight mental aberration, and the restricted responsibility as well as the peculiar kind of penalty, which should attach to a partial lesion—to which shortly I shall again refer—much weight will be given to your moral assistance; and apart from the particular circumstances of the case, he will have but to express an opinion not hastily formed in the midst of the excitement of a law court, but which you have deliberately conceived, and which will be stamped with the liberal, sagacious, and elevated character which your discussions indicate.

Considered from a psychological point of view, man is endowed with sensibility, intelligence, and activity.

From sensibility are derived sensation (pain and pleasure), feeling (fear and desire), and the passion which consists in energy or the exaggeration of feeling.

Sensibility enters, to a certain extent, into our actions, but is neither free nor enlightened.

Intelligence, very variable in degree, may be of a low or high order; it moves the mind of ordinary capacity, it inspires the man of thought, and illuminates the man of genius. But the point of distinction which characterizes the being endowed with intelligence, is that he bears within himself the idea of good and evil, of the just and the unjust.

Reason is the faculty which holds the highest position in our intelligence. It is that faculty which, whenever sensibility urges us into action, apprehends and estimates the morality of the act which we are about to perform.

With respect to activity, it consists in the resolution to do or not to do, it holds command over the organs, and is ordinarily expressed by the two terms, Liberty and Will.

Man then may become the primary cause of an act ; he knows the moral weight which attaches to it, and if its accomplishment be in opposition to that which is right, he renders himself chargeable for it. Now the chargeability of an act leads us to become answerable for it, and hence responsibility.

It sometimes happens that an action is performed under the influence of sensibility alone, without the interposition of intellect and will. We then denominate the action *instinctive,* or *irrational.* When an overpowering impulse of sensibility has not allowed to reason sufficient time to throw light upon the action performed, the term *spontaneous* is then applied ; and when the action is not accomplished except after examination and mature deliberation, it is termed *reflective.*

The amount of culpability depends upon these three degrees; in correspondence with these is a scale of punishability. In the instinctive or irrational action there is no chargeability ; in the spontaneous action there is chargeability, with a less degree of culpability ; in the reflective action there is entire culpability.

With these introductory propositions, let us consider what relations exist between psychical deterioration and the conditions of chargeability and culpability.

The legislator, unable to enter into all the details of cerebral pathology, has been most unwilling to recognise the unsettled technical terms employed by medical men. The magistrates, on their part, have rejected all innovations in the matter of nomenclature, and have adhered to their traditional classification. The terms *dementia, imbecility, furor,* in common use in courts at the present time, are found in the most ancient records.

D'Aguesseau, in the case of the Abbé d'Orleans, has applied to lunatics and imbeciles the following definition : "They suffer but a simple privation of reason ; the weakness of their mental faculties, mental perturbation and unsteadiness, the almost continual inconstancy of their minds, places their reason under a kind of perpetual suspension and interdiction, whereby they receive the name of *mente cepti.*"

The term *furor,* or mania, continues to designate, in legal phraseology, a general state of disorder, of perturbation of the faculties extending to all sorts of objects, characterized by incoherence of ideas, sometimes accompanied by illusion of the senses and hallucinations, and always attended with excitement.

In law the three preceding conditions have been admitted with one accord, and no exception can therefore be taken. How can we arraign before a tribunal of justice a madman, an idiot, or a maniac? To what sentence can we subject him? It is not exclusively to the French code that we must render this homage, but to the laws of most European nations besides, and even to the legislative texts of Lousiana. If it should have happened that the law has brought down her sword upon the head of any one devoid of reason, undoubtedly it is much to be deplored; but there is good reason to suppose that a similar error could not be committed in the present day.

In making use of the expression dementia, as synonymous with madness or mental aberration, our legislators have committed the grave error of not having defined it; they have thus abandoned this medical question to weak interpretations. Meanwhile, and as if good could result from an omission, it has become possible, by means of this, perhaps intentional, oversight, to give to the expression *dementia* a latitude sufficiently great.

Amongst the excesses of passion which outrage society, there are some of which the extravagance, infamy, and cruelty are so unwonted, that the law does not assert her authority except after mature deliberation. Between the simple declaration of affection, for instance, and sexual appetites of the most inflammatory description, there is a broad interval in which the emotion of love may more or less predominate, leaving to man an entire liberty of moral action, a restricted action, or perhaps an action altogether without power of moral restraint. If these minutiæ are not inscribed in our codes, they should be in the mind of the experienced medical man. In order that no error may be made, his function is to establish these niceties of differential diagnosis, making clear what judicial action ought to follow; but it ought not to consist in the philanthropic demonstration of an inexhaustible indulgence. Does not probity forbid us to justify immorality and place it in the same category with misfortune?

If the question of free will could be raised with reference to erotism, satyriasis, and nymphomania, with how much more force will it present itself in conjunction with two nervous diseases which partially pervert the human understanding—I mean hysteria and epilepsy?

In a recent publication, full of the most curious and carefully-observed matters of interest, Dr. Constans has not hesitated to represent the greater part of the hysterical patients at Morzines as being absolutely irresponsible for their actions. Now an affection which is but the expression of a peculiar susceptibility of the nervous system, and not a mental disease, can very rarely overpower moral liberty and exclude all culpability. Hysteria shakes the cerebral edifice, exercises a powerful influence, if you will, over the emotional faculties, and sometimes ends by inducting a true lesion, but ordinarily the intellectual faculties remain intact. The reason assists in the overthrow of the mind, but she survives it.

Therefore, as I once declared in a celebrated lawsuit which was terminated by an incomprehensible acquittal :—" The only cases of hysteria which, in my opinion, justify the application of the 64th article of the penal code, are those which are not unfrequently met with amongst young girls or women with whom this melancholy tendency is hereditary, who inevitably approach, and in a very short time arrive at, a state of complete madness, who in infancy have been specially subject to nervous and convulsive disorders, who have arrived at a period when the development of the intellectual faculties is arrested, and who, above all, spring from a stock in which are numbered many deranged persons." M. Tardieu has cited a very conclusive case of this description. That learned professor observed, eleven years ago, together with MM. Calmeil and Lasègue, a young woman of singular beauty, a member of one of the highest families of the Austrian aristocracy, who prostituted herself to every comer, even to men of the very lowest condition, and who ended by decapitating her newly-born infant, without in any way being conscious of her criminal act. Such instances are altogether exceptional; and without having seen the possessed of Morzines, I apprehend that my honourable friend, M. Constans, has allowed himself to be carried a little too far in invoking the extreme clemency of the law in their behalf.

Scarcely a year ago M. Trousseau came forward to proclaim from the floor of the theatre of the Academy that it was incumbent upon men of science *to snatch from the scaffold a large number of epileptics who had been adjudged criminals;* and he has propagated with much energy the doctrine of irresponsibility in cases of epilepsy. M. Trousseau, however, has unfortunately propounded a medico-legal error, for by no means every person suffering from epilepsy is of unsound mind; only, amongst a large number or persons so affected, the harmony of moral sentiments is broken, the character of the affections perverted, and the order of the senses disturbed. Madness is foreshadowed, but it is not necessarily produced. The epileptic, in a word, is only a *candidate* for mental alienation.

The physicians who are most eminent in the treatment of the insane, and who have the care of a number of epileptics, are in general very much disposed to extend, beyond measure, the sphere of irresponsibility in favour of these latter. One can understand perfectly well this tendency, for their patients only present at intervals uncertain transient glimmerings of reason; but we come into contact daily wherever we go with a class of epileptics in whom their terrible disease is compatible with soundness of intellect. The theatre of human affairs is open to their free action, and sometimes they attain to a position of considerable distinction. In case of a judicial sentence falling on one of them I leave you to consider how the theory of exoneration from punishment would then be received.

In the intervals of their attacks epileptics frequently have long periods of restoration to reason. Without doubt they remain egoists, suspicious, self-willed, irritable, passionate; it is equally true that they are contentious, without ordinary affection, causelessly querulous, captious, they sow discord and give way to hatred; but this is their natural disposition, and their unhappy temperament does not render them the less liable to be in a large number of instances held partially responsible for their actions. M. Baillarger was right when he proposed, in his paper read before the Academy, an extenuation of responsibility in favour of these unhappy persons. We know that M. Delasiauve entirely concurs in this view of the subject.

In his excellent work upon the *Mental State of Epileptics*, M. Jules Falret has propounded a doctrine which is somewhat surprising: "Generally speaking," says he, "in a doubtful case one ought to incline the balance in favour of validity of action, whenever the point is discussed in civil cases, whereas it should be inclined in favour of irresponsibility in criminal cases." I must confess that I cannot well understand this plastic interpretation, nor do I see how the civil actions of an epileptic can afford evidence of soundness of mind, which his criminal impulses have all at once discovered to be completely absent. There is here, in my opinion, a direct contradiction.

When a physician allows his conscience and his feelings to glide smoothly over the declivity of irresponsibility, he can go to a great extreme. It is thus that Professor Joire (of Lille), chief physician to the lunatic asylum of Lommelet, expresses, in a little work recently published,* certain medico-legal opinions upon drunkenness very much to be regretted. According to our honourable friend, society has no right to hold the drunkard responsible for the outrages he has committed. "Such a being," says he, "has lost his moral freedom, he is nothing more than a madman, he cannot then be held responsible for his actions; these have been performed while he had lost the free

* *De l'ivrognerie considérée comme forme de Folie Suicide.* Lille: 1863. Broch. de 52 pages.

possession of his intellectual faculties." M. Joire submits to no check in so favourable a course, and, after having likened the drunkard to a man temporarily insane and to a suicidal lunatic, he demands very appropriately that *he shall be placed in a position in which it will be impossible for him to indulge his irresistible passion, and be looked upon not so much as a culprit upon whom society has already too hardly pressed by its chastisements, but as a sick person whose recovery is to be hoped for.*

What are we to think of this proposition for the confinement of drunkards? The institutions for the insane were never intended to become refuges open to intemperance; no more are they, as others have endeavoured to show, silent and close dungeons; and if, since the Revolution of 1789, it has happened that men have been locked up for having rendered themselves obnoxious to the authorities, or for having appended their signatures to documents filled with obscenities, contrary to religion and offensive to the sovereign, it is now absolutely impossible, thanks to the law of the 30th June, 1838, that an institution for the recovery of health should ever become an ambush, a foretaste of the tomb, or a state prison.

With respect to my personal opinions upon drunkenness, they are as follows:—The inveterate abuse of alcoholic liquors should continue almost entirely without influence over responsibility, until there is manifested a confirmed and persistent mania. Habitual drunkenness ought neither to augment nor extenuate the consequences of the act committed, but it may to a considerable extent diminish or altogether do away with the suspicion that the immediate drunkenness has been contracted for a culpable end. It is with difficulty one can understand that the habit of getting drunk should become, on the part of magistrates, an object of gracious consideration when their office is to repress scandal and to punish immorality.

Maine de Biran maintains that the lunatic should be removed from the category of moral and intelligent beings. To him, the madman is nothing more than "an automaton, which ceases to be man in ceasing to be a being of free action, a machine alternately tranquil or furious, weak or vigorous, delirious or subdued, successively imbecile, enlightened, stupid, noisy, taciturn, lethargic, active, living, dead." Maine de Biran's opinion is clearly overstrained. A most objectionable habit of never recognising the madman as a fellow-being exists in the world: it is wrongfully supposed that he constantly dwells upon the illusions of his fantasy. Nothing of the kind exists.

A man affected with *partial insanity*—that is to say, with a number of delusions which, being limited to certain points, do not necessarily exclude the possibility of rationality on other matters—yields to the impulse of some unusual propensity. Ought we to conclude that the partial cerebral lesion has subverted his reason to such an extent that amongst all the acts which he has committed, we cannot frequently leave some one or other of them under his charge? Shall we deny to him all power of discrimination, when the criminal act is clearly remote from his habitual aberrations? Even though he possess the most sound notions with regard to the habits of life, and the obligations due to society, even though the fear of punishment has put an effectual restraint upon him, we guarantee to him, nevertheless, perfect impunity: he shelters himself then behind the inexhaustible clemency of men, whilst society offers herself voluntarily, and without any defence, to all his machinations.

Permit me here to quote what has been said by M. Delasiauve, in his excellent work on *pseudo-monomania,* with reference to the distrust which the evidence of medical men sometimes excites: —"In cases where there is an apparent power of discrimination," says he, "the magistrates not unfrequently find it difficult to grant an acquittal for a crime committed under the impulse of abnormal instigation, because they believe in the power of resistance, and medical men would that, for some fugitive apprehensions, without any marked influence over the ordinary determinations, having been apparent one day and effaced the next, bearing no relation to psychical derangement, their severity should be arrested in favour of misdeeds committed with a will ostensibly perverted. Is this admissible? Is it not preferable, that instead of offering violence to their conscience by certain objectionable dogmas, legitimate satisfaction should be offered to their scruples by means of prudent limitations?"*

The distinguished professor of medical jurisprudence at Berlin has given expression to some severe remarks upon what he calls "the ultra-philanthropic and absurd theory which consists in the admission that monomaniacs cannot be held responsible because the healthy portion of their intellect might have become sympathetically affected. We see," says Casper, "that thousands of monomaniacs have continued in the same state throughout their whole lives, without giving evidence of any general reaction, without being able to throw off their fixed idea; they are, meanwhile, masters of it, they recognise it as such, even laughing at themselves while acknowledging it; and, what is of even greater importance for diagnosis, *not unfrequently they will even assent to that which is raised in opposition to their fixed idea.* Such men clearly must be held responsible for

---

* *Annales Medico-Psychologiques,* 1859, p. 228.

their actions committed even in virtue of their fixed idea. But when the fixed idea has taken deep root in their minds—when, ceasing to be an habitual trifle of the imagination, it urges the patient into the dangerous current of a violent passion, such as self-love, rage, jealousy, and induces him to commit some criminal action, we can then admit that he has no longer power of self-restraint, and the patient must be considered as a general lunatic."*

Clearly, the Prussian medical jurist is wrong as to the consequences of an act committed under the influence of a fixed idea; and I regret so much the more this error, inasmuch as the great authority in this branch of science which he enjoys, has been able to influence judicial decisions to a very grievous extent. But, together with our learned colleague M. Brierre de Boismont,† I agree almost entirely with the opinion of Casper in respect to offences or crimes committed apart from the conceptions of mania.

The laws now in operation in Great Britain admit the responsibility to a greater or less degree of the monomaniac. They recognise, moreover, the capability for civil action of a person so affected, of which I will here cite a most extraordinary instance. An Englishman who, it is said, had throughout his whole life been considered to be of sound mind, bequeathed a large part of his fortune to his landlord, on condition that he should make into violin-strings the intestines of the testator, and with the remainder of his body, crystallized glasses for optical purposes. He added: "I know that I shall be treated as a person of eccentric mind, but I have an inveterate antipathy for the funereal equipage, and I wish that my body may serve to some useful purpose." The will was disputed by the natural heirs, but, in accordance with the English code of law, it was pronounced valid.

In the case of an individual whose intellect is only touched, ought we to allow, generally speaking, that he has not power to oppose a sufficient and adequate resistance to his morbid suggestions, and that immunity from penal liability should, as a matter of course, be awarded to him? I think not, because the patient who is affected to this extent is not entirely carried away by his madness, and a certain number of his actions bear the mark of the freedom of his will.

You will perhaps recal an incident which happened to M. Delasiauve. This learned colleague found himself one day during an hour at table in an asylum, by the side of a lady

* *Traité Pratique de Médecine Légale*, t. i. p. 351.

† *Annales d'Hygiène et de Médecine Légale*, Octobre, 1862, p. 450.

afflicted with monomania. M. Delasiauve had been previously made acquainted with the fact; nevertheless, he could not but admire "the brilliancy of an intellect so animated, and the evidence of so finished an education. When narrating this anecdote, the distinguished doctor of Bicêtre gave utterance to this opinion, which carries great weight. "They may ramble upon one point, and preserve a correct reasoning upon others, surrender themselves, in matters which relate to their delusions, to whimsical actions, without, in other respects, offering offence to the conventionalities of society."*

The motive which leads us to take a part in the consideration of criminal affairs, is to analyse such actions as render a man responsible, and to determine, as far as possible, the extent of the power of reasoning which remains to the accused at the time of the accomplishment of the offence or perpetration of the crime. *Non creditur testibus de furore deponentibus, nisi causam reddant scientiæ.*†

The medical jurist ought then to be conversant with the degree of enchainment of the moral power, to weigh the more or less unwonted and extraordinary nature of the act committed, and to perform a judicious analysis of the faculties of intelligence. These characteristics should be clearly discernible in his answers to the questions which are put to him by the magistrate, and which are ordinarily expressed in terms analogous to the following. At the moment when such a thing was done, did the defendant possess the knowledge of right and wrong? Was he in possession of his freedom of moral action? Were these two faculties impaired when he performed the action in question? Was there not some impairment or disorder of intellect in other respects, or might this not have been the case with regard to his consciousness? What, then, was the nature and extent of this impairment? &c. &c.

I must confess that I do not clearly comprehend mathematical limitations relative to clinical matters, neither can I conceive how the doctrines of responsibility and irresponsibility have been so constantly opposed, the one to the other, without some mutual harmony having supervened.

It will be asked how we can lay down the invariable laws and determine the precise and persistent limits of health and disease? Do we not see, day after day, men of sound mind, who give evidence, in their occupations and pursuits, of divers degrees of understanding, presenting the most striking contrasts to one another? One man possesses an extraordinary power of

---

* *Journal de Médecine Mentale*, p. 360.

† Boerius, Dec. 23, No. 44.

memory, but is deficient in judgment; another is endowed with a most fertile imagination, but his force of will is notoriously restricted; one, endowed with all the bounties of intellect, by his impropriety offends against all the conventionalities of society; another, whose genius no one will wish to deny, lives in a barbarous solitude, &c. &c. Must we not, in like manner, award their due influence to instincts, affections, and passions which instigate men to commit actions difficult to analyse psychologically, more difficult still to classify in the science of medical jurisprudence? The answer to this question does not seem to me to present any great difficulty: the man commences to become a person of unsound mind when he begins to differ from himself.

With the geometrical line of demarcation which has been proposed and which very many have adopted, where will you range that mixed category of beings with whom our state prisons are for the most part filled—those individuals who having given themselves over to debauchery, have become permanently divorced from morality and conscience, and passive witnesses to their forfeiture, allow themselves to fall without resistance from vice to delinquency, and from delinquency to crime? Their nervous system is enfeebled, their perception obscured; here is a point for diagnosis, and inasmuch as these men must either be held to be of sound or unsound mind, the judicial consequences are the most simple, and end either by a condemnation in all its severity, or by an acquittal! For these modified (*mixtes*) beings, as I have denominated them, we must have recourse also to a modified means of repression. To the partial affection of the mind, let us oppose, then, a penalty of a special order.

Do you call to mind what occurred at Augsburg between 1817 and 1825? Well! five girls were wounded in nocturnal attacks. Charles Bentle, aged thirty-seven years, confessed to having wounded all of them, and declared that he had taken every precaution not to wound them dangerously. His plea was that he had been instigated by an "irresistible impulse." Seven daggers were found at his house. The Court returned him guilty, and sentenced him to four years' imprisonment. Here we have a penalty clearly mitigated; the judgment was reasonable, but nevertheless I cannot accord my unreserved assent.

Can we apply to the passions the same rules as we can to madness? Evidently not. Violent passions influence the judgment, and even throw a false colouring over it in a grievous manner, but they cannot annihilate it; they occasionally carry away the mind to form extravagant resolutions, but on the brain no pathological trace can be detected. I will grant that in the instances we are discussing they might override the will, but moral responsibility is only lessened but not removed. The penalty is only mitigated: to a graduation of culpability corresponds a graduation of liability.

Why not admit, indeed, a distinction between a murder premeditated, planned, committed in cold blood, induced by hatred, by vengeance, or by cupidity, and the assassination consummated on the spur of the moment, and under the impulse of jealousy without restraint, or of an overwhelming provocation?

By means of attenuating circumstances, and from the multitude of shades which the human passions reflect, the reasons for extenuation are gathered from the cause and with regard to the moral contest of the agent with himself. The law has been far-seeing and generous, but there is wanting some limitation of its bounty; nor do I think that such should any longer be held back.

I am surprised to find that some distinguished minds have been able to look upon partial responsibility as an enormity. "What would become of us," writes Dr. Belloc, "we who have the care of the insane, should the doctrines of absolute irresponsibility be put in force for a moment in an asylum? Is not our whole influence, our whole power of government based upon the capability of the lunatic to comprehend the admonitions which are given to him, the reprimands which are addressed to him, and to regulate himself in accordance? Every day," adds he, "in the asylum which I superintend, I command, I reward, I blame, I enjoin, I constrain, I threaten, and I punish. And in the presence of these facts, what becomes of the doctrine of entire irresponsibility which we afterwards maintain before the courts of justice? I can only reconcile this flagrant contradiction to myself by the spectre of the guillotine which the public minister never tires of presenting to the public gaze. In the presence of such a danger as that threatening one of our insane patients, we have thought that nothing was too much to do, and we have in consequence imperceptibly overstepped the limits of reason and justice."* I myself altogether agree with the opinions expressed by our honourable colleague d'Alençon. Yes, in a case suggested, the reason may be partially impaired, but the morbid lesion is isolated; the psychical key-board has some false note.

Since, then, we recognise amongst certain of our lunatic patients a variable, but no less certain, amount of intellect and moral freedom, in whatever way these partial attributes are regulated, to whatever end they are applied, in whatever conditions they are brought into use; is it not then possible to analyse

---

* *Annales Médico-Psychologiques,* 1861, p. 422.

these sorts of modified states, to separate contrarieties and to elucidate the details. It will be objected that even though some rational ideas shall still lend their impression to a diseased brain, the man is none the less a living and harmonious unity; that it is impossible to parcel out the mind; that in our psychological organism there is nothing independent, nothing fragmentary, and that amongst the different faculties there exists a principle of succession and inter-connexion which prevents their being isolated; that it is impossible to reckon the grades by which reason glides down the precipice, and so forth. I foresee all these arguments, but I do not believe in the absolute interdependence of the faculties, and I rest convinced that there may be absence of reason, absence of knowledge of right and wrong relatively to certain objects, without there being with respect to others any visible alteration of the understanding. I believe, moreover, that the delusion is sometimes so localized, so circumscribed, and that the mind is so free in all other respects, that the lunatic appears to be of sound mind as long as his attention is not directed to that point upon which he has delusions.

During six weeks I have travelled along with a man forty years of age, of superior intelligence, of sound education, and of remarkable learning, who, under the influence of hallucination of hearing, had made use of the most various and terrible means to rid himself of life. He had always recovered from his wounds! My fellow-traveller enjoyed all the worldly goods and all the comforts which could insure a happy existence; it seemed even that he possessed everything that he could desire. I encouraged, during our travels, his inclinations and tastes for archæology and painting, and I listened with much interest to his observations upon the fine arts. We followed the same pursuits more than forty days, and we carried on long conversations upon all possible varieties of subjects; he often attacked me upon the subject of madness, and he was surprised that I endeavoured always to bring about some incident for the purpose of at once diverting the line of conversation. "I have read," said he, "the works of Esquirol and MM. Morel and Brierre de Boismont; ah, well! it seems to me that these doctors have not made out a very clear case on the point of liberty of will. As far as I am concerned, if I were to inflict a sound correction on one of those individuals who now and then venture to insult one grossly in the streets, I could not be held responsible for it, for the desire to avenge myself for these insults would have blinded my judgment; but if I were to come up to you and seize upon your purse, I should be nothing but a thief; and here lies all the difference." The patient managed habitually to conceal his hallucinations, but the profound distress which they caused him inwardly, always ended by leading him to commit against himself some new act in very despair.

Let us not then exhaust ourselves in superfluous efforts to establish dogmatically that such an one is guilty or innocent, that he is of unsound or sound mind; there remains very frequently, in fact, an intermediate position, which after deliberate examination, permits us to decide that some portion of the faculties of the mind remain unshaken. Let us have the frankness to acknowledge this, let us learn to pronounce in a given case how far a partial lunatic may rest a stranger to the perpetration of crime, and then shall we commence, physician and jurists, to speak the same language, to the inestimable benefit of both science and humanity. Then shall we no longer behold the courts of justice present to the world's gaze the absurd spectacle of their contradictions and Justice with her balance replaced by the wheel of fortune. Our evidence will no longer, as at present, be received with distrust, and science will have laid down with mature consideration and wisdom the most equitable solution of a debated question. When we see judges, whenever it becomes necessary to verify the genuineness of a document, placing no confidence in their own powers of observation, but calling to their assistance experts, it will be readily understood how gladly they would receive enlightenment from us, upon the obscure or complex symptoms of a wandering intellect. Is not their repugnance to admitting medical depositions fairly justified by our tendencies to exaggeration.

Having reached this point of the discussion, I hasten to approach the question of penality; for the preceding observations have not in any way given intimation of the measures which it still remains for me to propose, after other physicians of greater authority. In premising that certain lunatics are to be held responsible, under definite restrictions, for the morality of their actions, this is by no means to the intent that, after having incurred a penalty more or less insignificant, these persons of unsound mind should in consequence be subjected to the miserable confinement of a prison. The solitary system, which has so wrongfully been adopted in France, and which is recognised to a certain extent as a cause of insanity, would in a very short time bring about the complete destruction of these frail intellects. No; in the matter of partial insanity, I am no advocate for the privileges of attenuating circumstances: the penal abatement diminishes the liability to criminal actions, but it leaves untouched the guilt, and the family of the culprit will inevitably hereafter bear the indelible impress of the judicial brand.

As I have already observed, in cases of dementia, criminal process is arrested; there is no crime to expiate, but a misfortune to prove. Punishment would be an injustice useless to society, because punishment is only inflicted by way of example; in cases therefore where an example cannot be made, punishment would become a barbarity. The bastinado publicly inflicted upon a fever patient would be far from curing the fever.

If discussion is to be held upon the restricted sphere of morbid action, I think that modified means of repression should be adopted, and that an additional clause might with beneficial results be appended to the Act of the 30th of June, 1838, directing the construction of a central establishment specially devoted to the accommodation of persons of unsound mind convicted for offences; or otherwise the opening, in the four principal asylums for the insane, of a special department. A criminal sentence would probably not rest upon these individuals; the impress of crime would by no means, as a matter of course, be set upon their forehead. Perplexities would thus be set at rest, all desirable guarantees would be given for the public security, and a restraint of this kind would prove a safeguard to the reputation of families.

In a very remarkable treatise, M. Brierre de Boismont proposed, seventeen years ago, and for the first time in France, the construction of an asylum for the vagrant convicted lunatics, and accused persons who simulate unsoundness of mind. The excellent arguments which he advanced at that time are even in the present day full of truth.

There exists, indeed, a class of harmless persons, wandering about in the public streets, with but very little intelligence, for the most part without occupation, living in idleness and want, and whom the offence of mendicity frequently brings before the police authorities. They are convicted, and at the expiration of their term they once more renew their *vagrancy*. They are again brought up before the police, and if their weakness of intellect is proved, they are sent to Bicêtre. After their entry they became quiet; and as they do not appear to be in any respect dangerous, their discharge is sought. Fresh difficulties are not long in supervening, their relapses are numerous, and these vagrants come before the criminal courts or are returned to the hospital a great number of times.

How many women there are of imperfect development of intellect, without power of self-restraint, the subjects of nervous disorders, who fall into degradation, and who in their improvidence—amounting to disease—have never had any other prospect than misery, shame, and suicide!

What is to be done with these individuals? Put them in prison? The contact with criminals will only render them more perverted. Place them in a lunatic asylum? They will suffer wrongfully by an association so unjust and inappropriate. Has not society a right to protest, on their behalf, against an admixture which is unauthorized both by the laws of morality and those of pathology?

As it has been shown by M. Brierre de Boismont, the provision for criminals of unsound mind which has existed for a long period in England is constantly giving rise to the very best results. The patients are there subjected to perfect observation, they are placed under strict surveillance, but at the same time treated with careful indulgence and humane attention; their time of probation is thus passed in the midst of quietude which would in vain be sought in the anxieties of a prison or confusion of a madhouse.

The department for criminals of unsound mind which exists at the hospital of Bicêtre is established upon the most unjustifiable principles, and it brings to mind too forcibly the incarcerations of times gone by to be worthy of our age. I simply refer to it, for it is most defective, established upon wrong principles, and is too little in harmony with our leading charitable institutions, to be for a moment taken into account.

Persons afflicted with partial unsoundness of mind, and who have committed such actions as would render them liable to punishment, should, after judicial inquiry, with medico-legal investigation, be placed in the central establishment or one of the special departments of the asylum designated, and the authorities, when determining the duration of their confinement, could estimate it from the length of penal servitude to which they have rendered themselves liable.

The State supports at great expense in the colonies, agricultural and penitential, twelve thousand children who have fallen into the hands of justice. These young culprits have committed crime, without discerning its nature, it is true, but their acquittal could only be carried out by means of confinement of greater or less duration in a special establishment. We should esteem it a happy thing if some system analogous to this were pursued in the case of the insane whose criminality is only partial.

The medical jurist entrusted with the examination of a criminal of unsound mind ought, before drawing up his report, to make the most careful investigation into the particulars of the life of the accused; analyse his previous actions, the habitual tendencies of his mind, and weigh all the psychical manifestations which have preceded, accompanied, and followed the criminal action. The best way of preventing the repetition of troublesome legal differences would be to fix some definite points of reference relative to the medical diagnosis of responsibility, and, from the advanced state of science in the present day, it is possible to enumerate them summarily, as follows:

1st. Is the crime an isolated fact in the life of the accused?

2nd. What have been the motives of it?

3rd. Did the defendant follow any systematic plan in the accomplishment of the deed of which he stands accused?

4th. Has he endeavoured to escape from punishment?

5th. Can any regret or penitence be detected?

6th. Can he render an account of all the details of the action?

7th. What were the peculiarities of his mental state, one or more years previously?

8th. Has the accused been at any time, and is he at present subject to hallucinations, and which are the affected senses?

The application of these eight elements of diagnosis is calculated very much to facilitate the task of the experienced physician, who, as a general rule, should never overstep his proper sphere. If he constitute himself an advocate, he loses all his influence over his audience, because the judge and counsel consider themselves more competent than he in that respect. He should elucidate the facts of the case in a scientific way, offer his opinion in a deliberate and decisive manner, making reference to analogous cases which have previously fallen under his observation. He will at times find himself placed, during the course of a law-suit, in a position of unforeseen difficulty, so that the perplexities of the moment may, at any time, plunge him into a state of the utmost embarrassment; but as there is between the physician and the ordinary witness the same difference which distinguishes the man of intellect and the man who is guided merely by his feeling, he will have to take counsel of his knowledge, his education, and his integrity. Thus represented, the cause of science will become also the cause of truth.

There is, lastly, one other question, on a point of legal interpretation, which presents itself for consideration. Ought the question of insanity to be proposed to a jury? The Court of Cassation has always replied in the negative. It is, however, evident that the text of the law does not prohibit this course. On what ground, moreover, if doubts concerning the complete soundness of intellect of the accused arise during an inquiry, should the jury maintain silence upon a question so much calculated to modify the responsibility of the agent? If the question be not proposed at all, juries will not suppose that it is a matter for their deliberation. A very great difficulty is thus created. If juries, on the other hand, fully convinced of the unsoundness of mind, return a verdict of Not guilty, are there no further measures to be taken by the authorities ?

My final view of the whole question is this:—We have a right to demand that our intervention in a law-suit in which a question of morbid psychology is to be decided, shall no longer, as previously, rest with the judge, nor depend upon the discretionary power of a president of assize. Our interference with civil or criminal affairs of this nature ought to be inscribed in our codes as the most indispensable of the formalities of the procedure. Society would then no more have to deplore those terrible verdicts which have deprived the insane of life, nor regret those unmeaning sentences which neither execute nor grant pardon, but which give evidence, by a compromise, of the painful alternatives of a doubtful judgment.

To recapitulate, the time has arrived for us to abandon, with respect to partial insanity, the too absolute doctrines of irresponsibility *in toto,* and to give greater authority to the motives which might mitigate a sentence, diminish sensibly its severity, or lead to the adoption of special measures, and supersede, in a word, a preponderating influence in the courts of justice. Do not, then, let our co-operation be wavering or doubtful, but let it be such as to gain universal support, by enlightening the convictions of all.

---

